# Supplementary figures and images for: Transcriptome profiling of barley and tomato shoot and root meristems unravels physiological variations underlying photoperiodic sensitivity
Source: PLoS One. 2022 Sep 12;17(9):e0265981. doi: 10.1371/journal.pone.0265981 (PMC9467324; doi:10.1371/journal.pone.0265981)

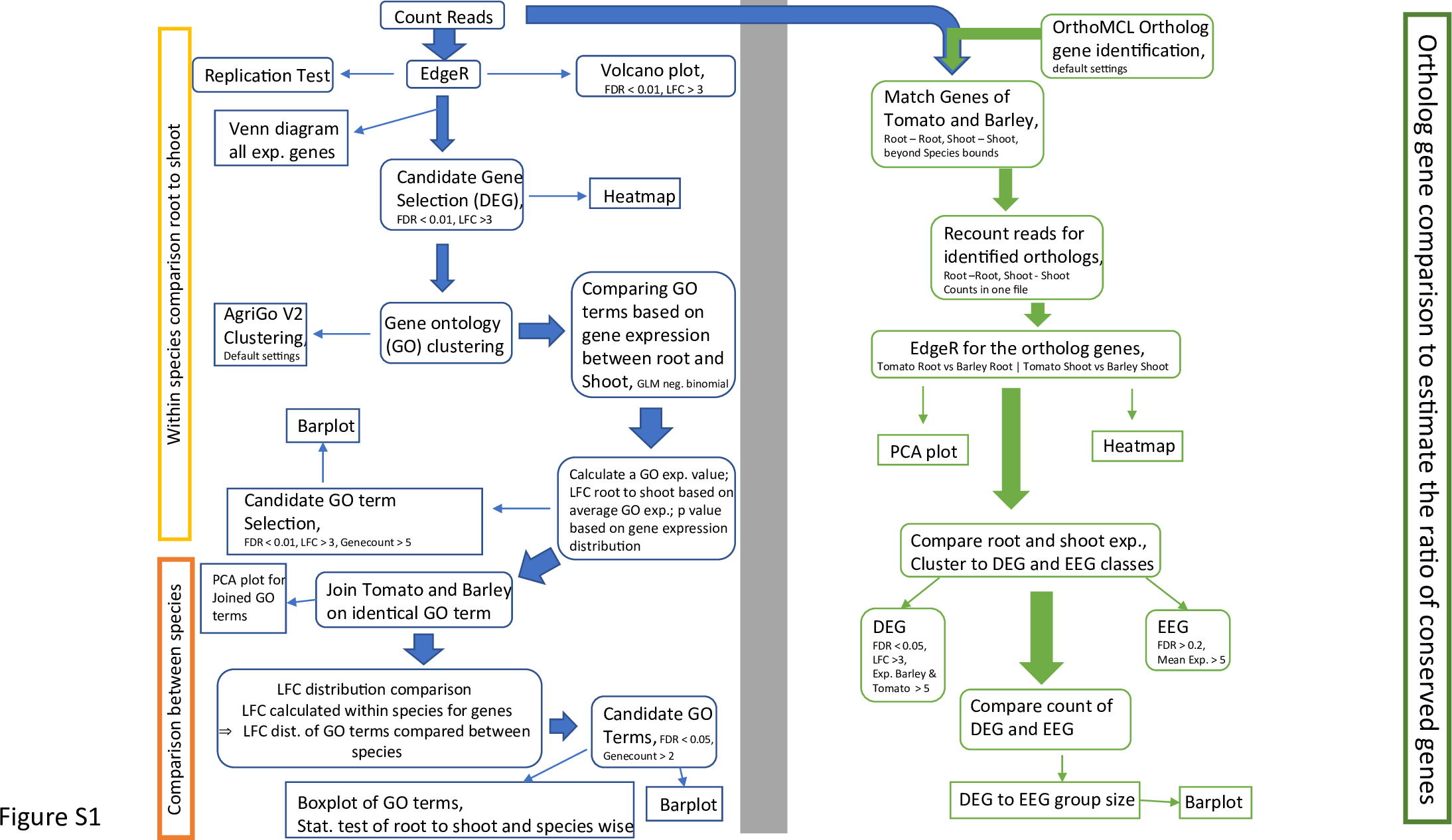

Supplement: S1 Fig — Each level is framed by a yellow, orange, or green square. (TIF) [file pone.0265981.s001.tif]

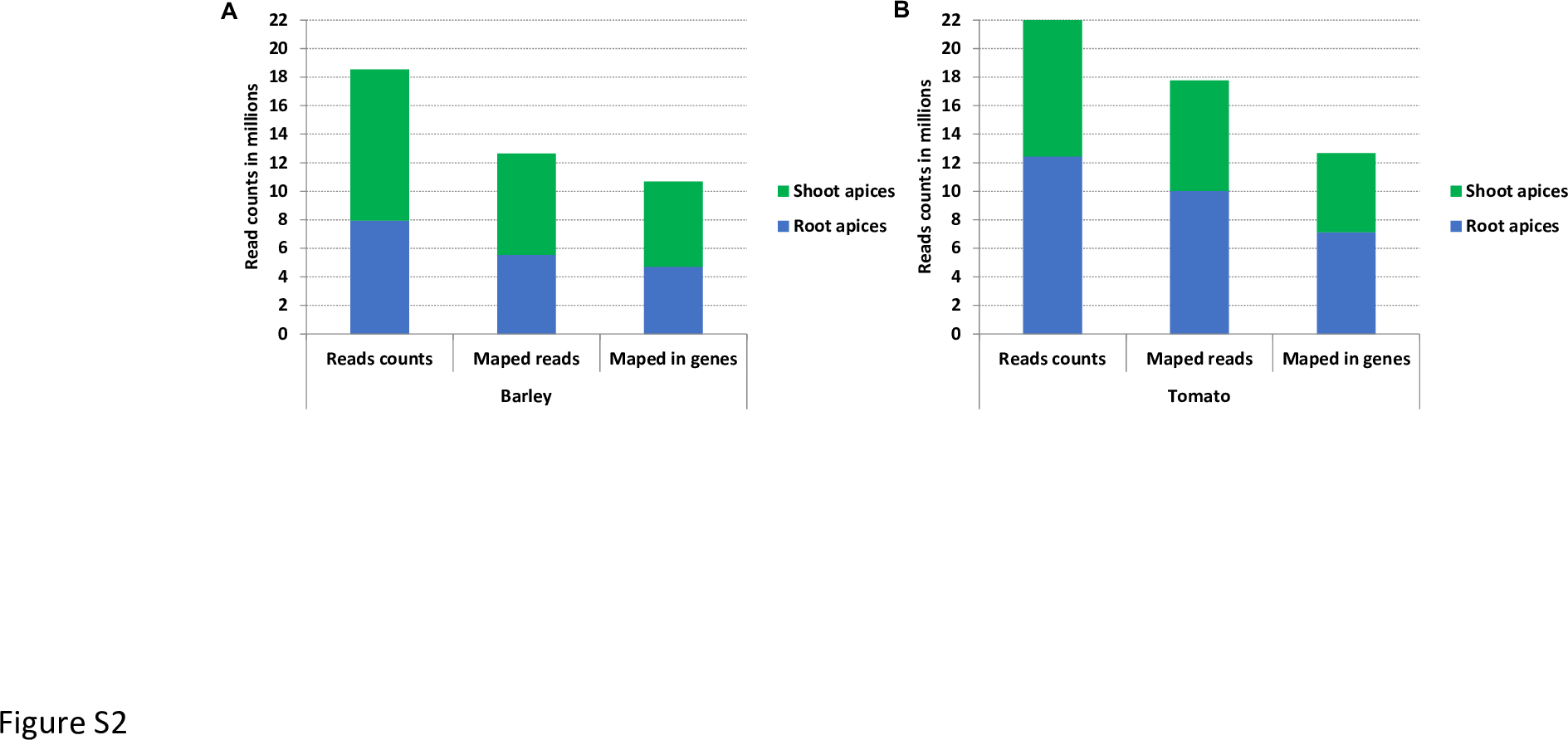

Supplement: S2 Fig — The number of reads (in millions), mapped reads, and mapped in genes in Barley (A) and tomato gene annotation (B). (TIF) [file pone.0265981.s002.tif]

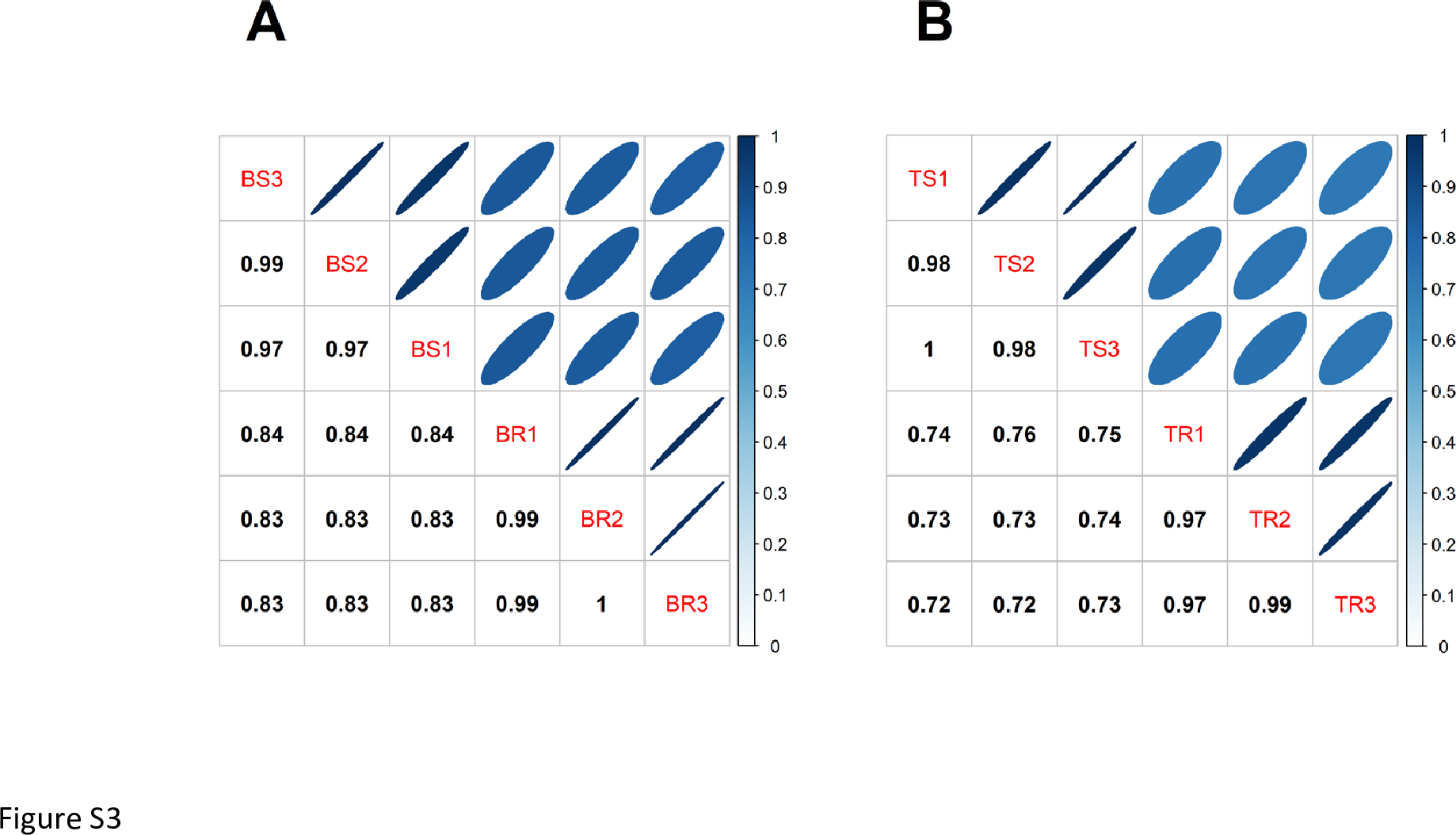

Supplement: S3 Fig — Correlations among three biological replicates of root apices (BRx / TRx) and shoot apices (BSx/ TSx) in Barley (A) and tomato (B). Correlation is illustrated by color, shape, and additionally as a numerical value. (TIF) [file pone.0265981.s003.tif]

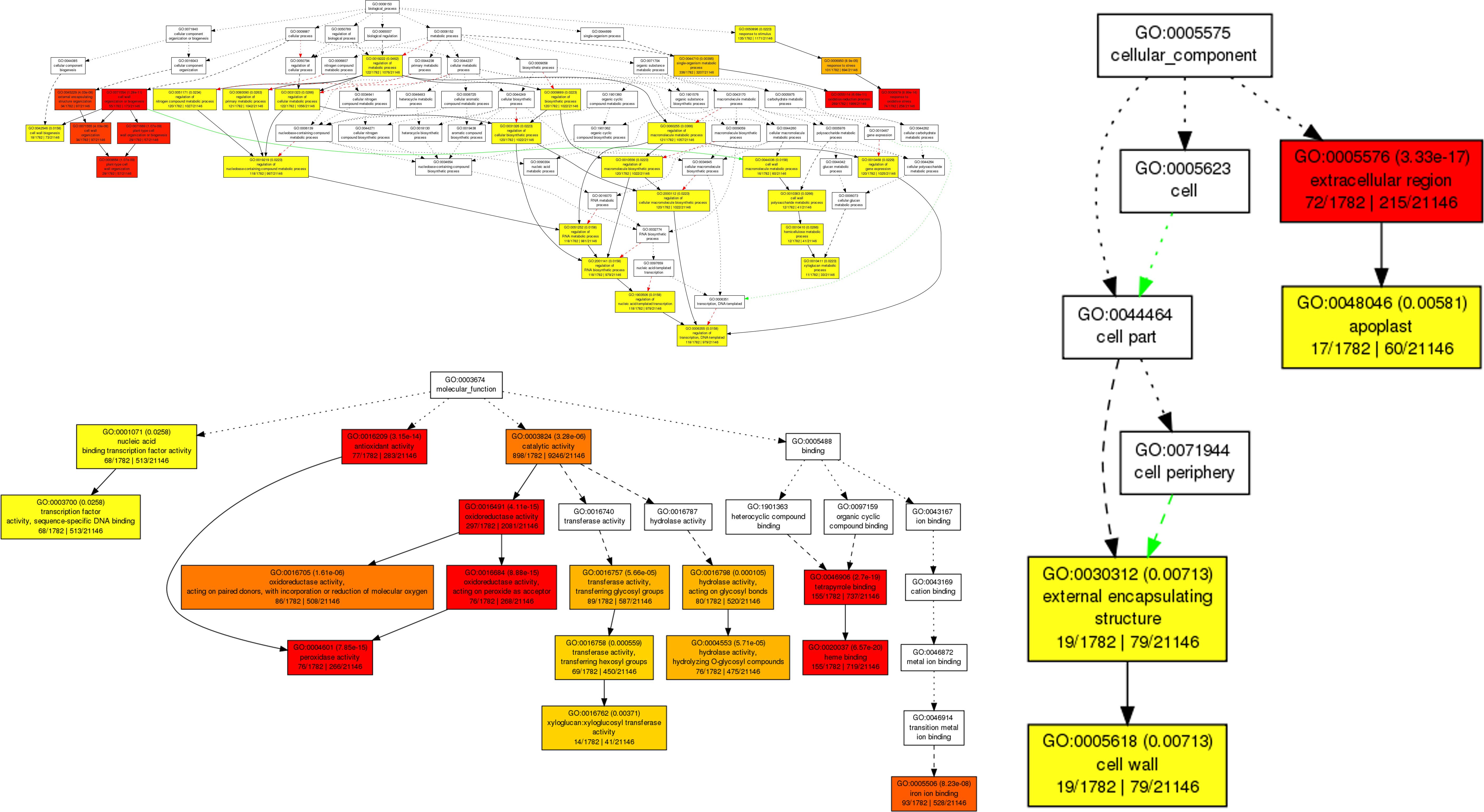

Supplement: S4 Fig — A–biological process; B–molecular function; C—cellular component. (TIFF) [file pone.0265981.s004.tiff]

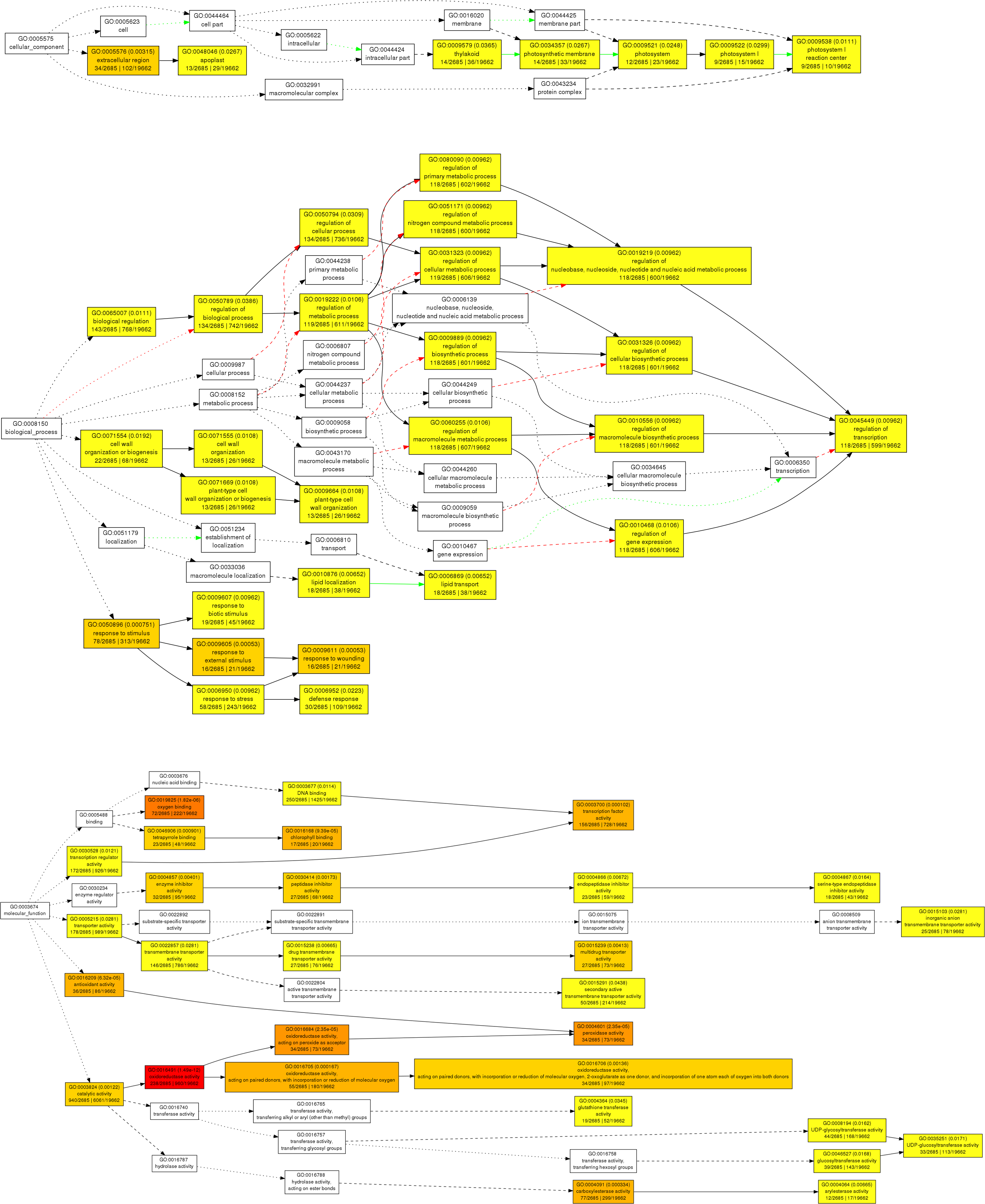

Supplement: S5 Fig — A–biological process; B–molecular function; C—cellular component. (TIFF) [file pone.0265981.s005.tiff]

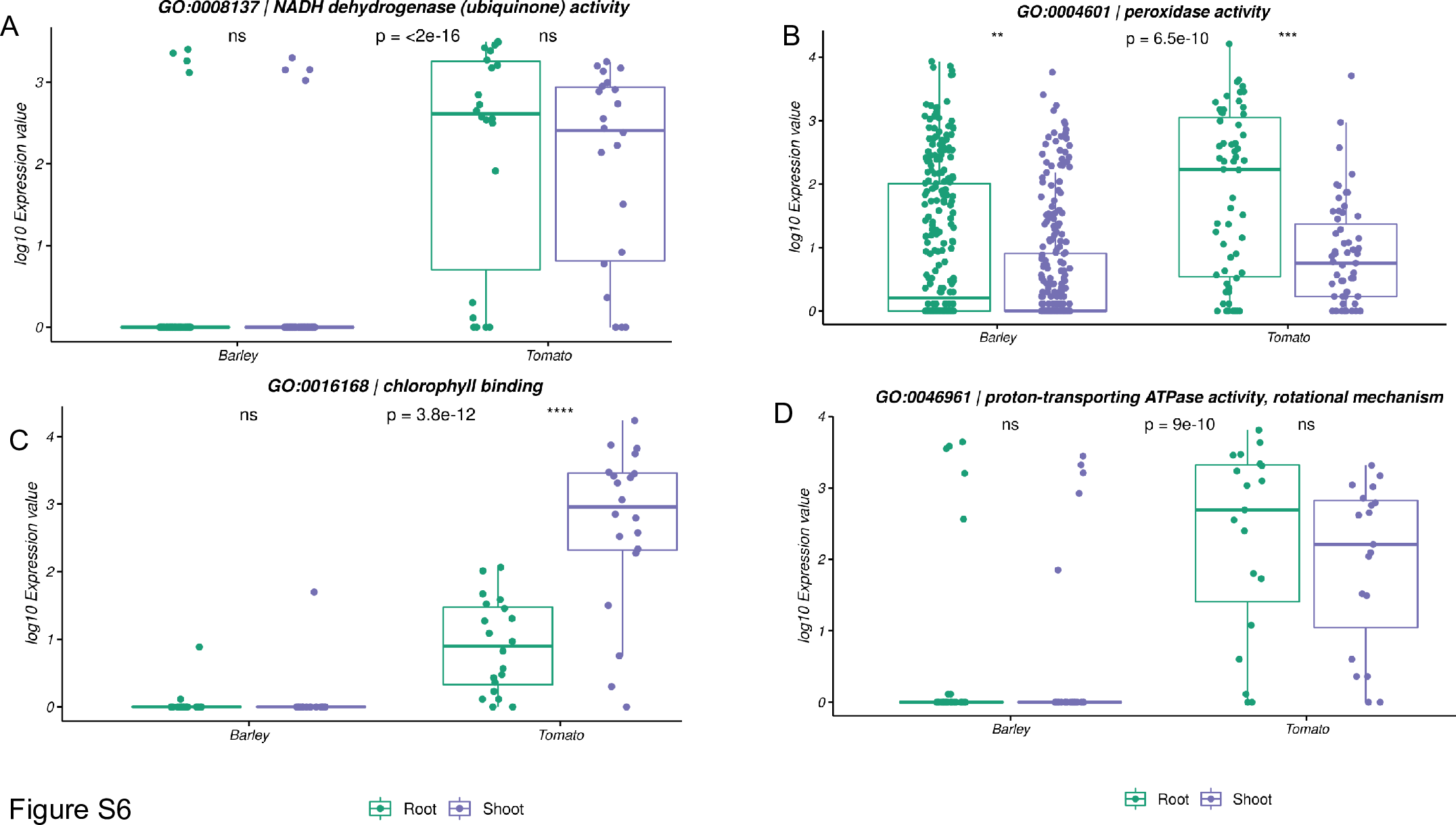

Supplement: S6 Fig — Each dot represents the expression level of a single gene, while the boxplot summarizes these single points. Root and shoot are separated by color, while the species are spatially divided. Two statistical tests were performed, between the tissues and between the species for each GO term individually. The results of the tissue-wise comparison are printed below the GO term name between the green and purple boxplots, while the species-related comparison is placed between the spatially separated boxplots. (TIF) [file pone.0265981.s006.tif]

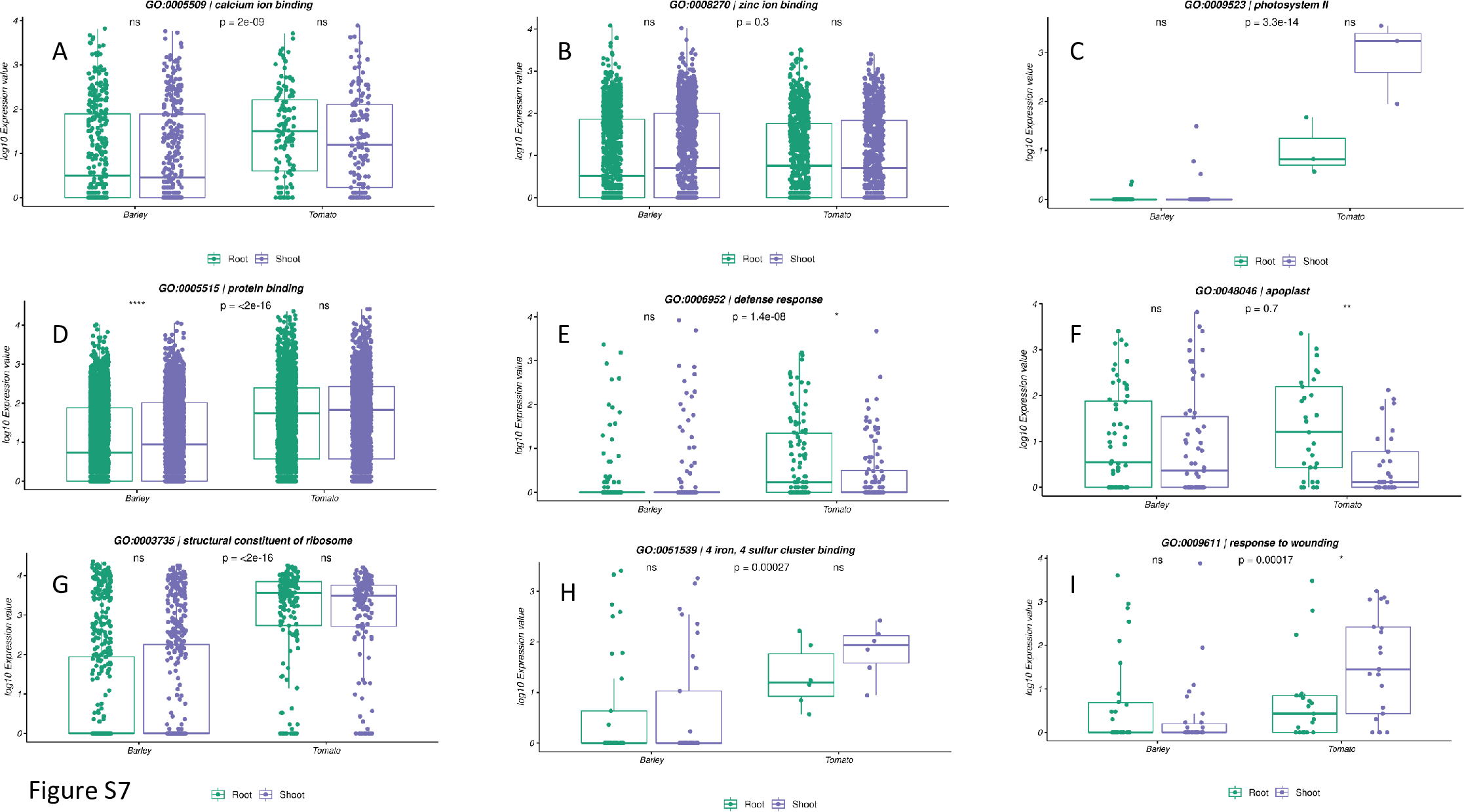

Supplement: S7 Fig — Each dot represents the expression level of a single gene, while the boxplot summarizes these single points. Root and shoot are separated by color, while the species are spatially divided. Two statistical tests were performed, between the tissues and between the species for each GO term individually. The results of the tissue-wise comparison are printed below the GO term name between the green and purple boxplots, while the species-related comparison is placed between the spatially separated boxplots. The GO term and function are illustrated above each boxplot. (TIF) [file pone.0265981.s007.tif]

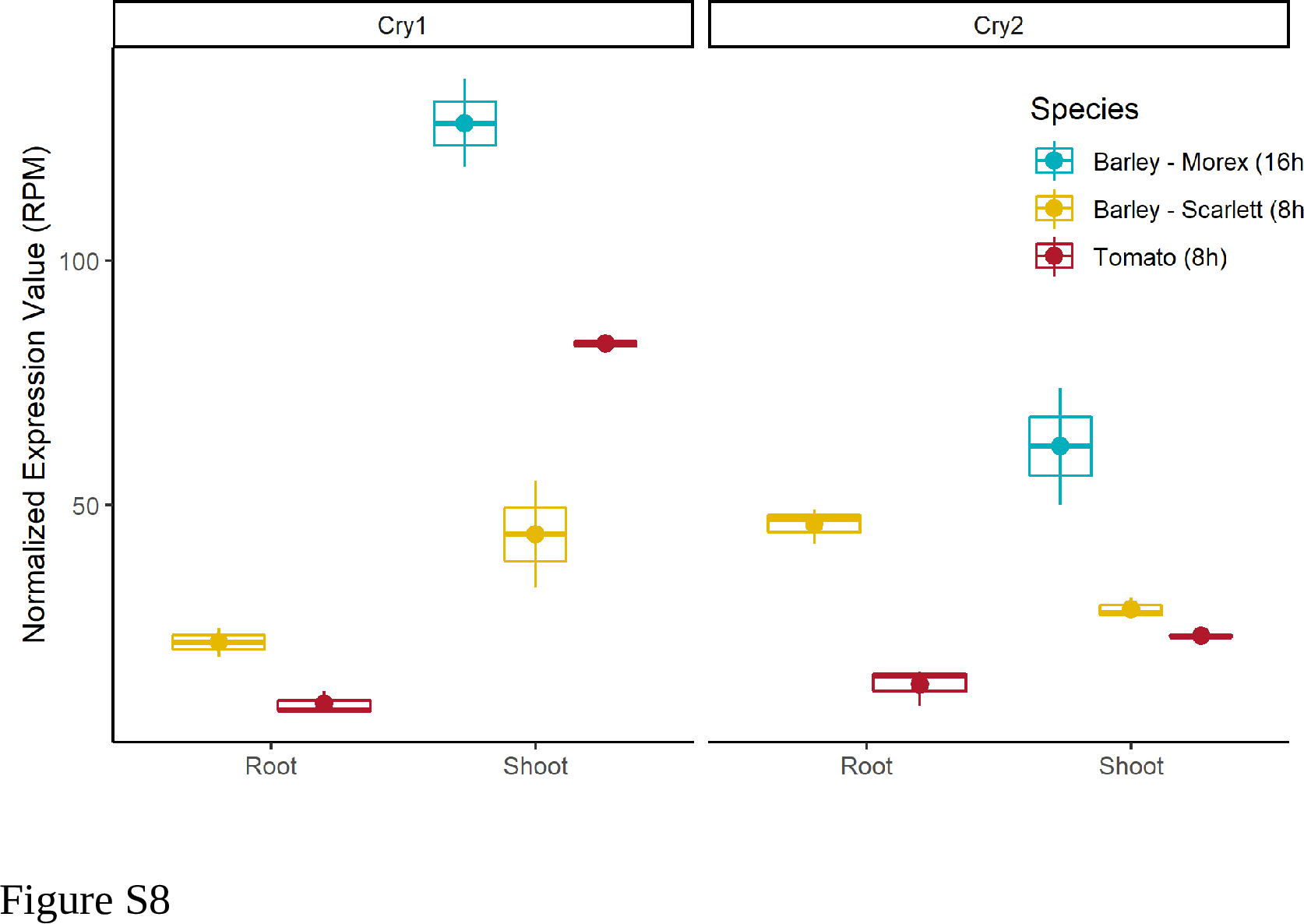

Supplement: S8 Fig — The expression is compared between tissues (x-axis) and species (color). Expression data for Morex, published by Liu et al. 2020 was added to compare barley expression patterns under short-day (Scarlett– 8h) and long day (Morex– 16h) photoperiods. (TIF) [file pone.0265981.s008.tif]
